# Gestational weight gain velocity during each trimester is critical for both maternal health and birth outcomes in China

**DOI:** 10.1111/mcn.13597

**Published:** 2024-02-28

**Authors:** Zhenyu Yang, Jianqiang Lai

**Affiliations:** ^1^ National Institute for Nutrition and Health Chinese Center for Disease Control and Prevention Beijing China; ^2^ Chinese Center for Disease Control and Prevention Beijing China

Obesity has become a global public health concern. The worldwide prevalence of overweight and obesity for child‐bearing women has dramatically increased over the past 40 years (NCD Risk Factor Collaboration, 2016). As the beginning part of the first 1000 days, pregnancy is a critical window for both maternal and child health in both the short and long term. Maternal nutrition can impact foetal intrauterine growth and development, low birth weight, macrosomia, and childhood obesity (Larque et al., 2019). In addition, maternal nutrition is associated with maternal complications during pregnancy, including inappropriate gestational weight gain (GWG), gestational hypertension, and gestational diabetes (Santos et al., 2019). GWG is a common indicator used to monitor nutritional status during pregnancy. GWG is significantly related to small‐for‐gestational‐age (SGA), preterm birth, large‐for‐gestational‐age (LGA), macrosomia, caesarean section, gestational diabetes and gestational hypertension (Goldstein et al., 2017; Santos et al., 2019). Excessive GWG is related to higher post‐partum weight retention and percentage of body fat, even at 7 years postpartum (Widen et al., 2015), which could increase the risk of overweight/obesity for child‐bearing women.

Inappropriate GWG has become highly prevalent. A meta‐analysis showed that 51% of pregnant women had excess GWG (EGWG) and about 20% (18% or 21%) of pregnant women had inadequate gestational weight gain in Europe and the USA (Goldstein et al., 2018). Based on Asian BMI criteria, a similar prevalence of inappropriate GWG was seen in Asia (Goldstein et al., 2018). According to a national survey in China, two thirds of pregnant women had inappropriate GWG based on IOM standards (Wang et al., 2018).

Several GWG guidelines have been developed to monitor weight gain during pregnancy (IOM Institute of Medicine and NRC National Research Council, 2009; Kac et al., 2021; Standard of Recommendation for Weight Gain during Pregnancy Period, 2022). Some scientific bodies across several developed countries, including Australia, New Zealand, and the United States, have adopted the Institute of Medicine (IOM) GWG guideline (Harrison et al., 2021), which is based on observational study findings from populations in high‐income countries. However, this guideline is not applicable to other populations including those that are thinner and shorter women compared to American women according to IOM's statements (IOM Institute of Medicine and NRC National Research Council, 2009). Few developing countries have established their own GWG guidelines. For example, a recent Brazil GWG chart was shown to be quite different from the IOM GWG chart, regarding the rate of GWG (Kac et al., 2021). In China, a GWG standard was released recently (Standard of Recommendation for Weight Gain during Pregnancy Period, 2022), which differs from IOM GWG standards in terms of total GWG (China: 8–14 kg vs. IOM: 11.5–16 kg) and GWG velocity during the second and third trimesters (China: 0.26–0.48 kg/wk vs. IOM: 0.35–0.50 kg/wk) for normal weight women. The World Health Organization (WHO) is calling for data on GWG to develop global GWG standards (World Health Organization, 2023), which could be applied to women of all pre‐pregnancy body mass index (BMI) levels and across different regions. The aim is that these optimal GWG standards would be used to improve maternal and infant health outcomes.

The purpose of this special collection of papers is to bring together research that has been conducted on GWG in China. These papers (Song et al., 2023; Wang et al., 2023; Yang et al., 2023) provide new local evidence that may be used for developing global GWG guidelines.

Previous studies have shown that the total GWG is associated with LGA and SGA (Goldstein et al., 2017), but few studies have assessed the relationship between GWG velocity during each trimester and birth outcomes and pregnancy complications (Zheng et al., 2019). The paper by Wang et al. (2023). in this section of *Maternal & Child Nutrition* is based on a prospective cohort study in China, in which the gestational weights were measured on average 10 times during women's pregnancies. The prevalence of LGA and SGA was 12% and 4%, respectively (Wang et al., 2023). For pregnant women with normal BMI (18.5–23.9 kg/m^2^), LGA newborns were associated with higher GWG velocities than appropriate‐for‐gestational‐age (AGA) newborns in each trimester (first, second and third, respectively). By contrast, SGA newborns were associated with lower GWG velocities than AGA newborns only in the second and third trimesters (Wang et al., 2023).

The second paper in this section describes the relationship between GWG timing (each trimester) and hypertensive disorders during pregnancy (i.e. gestational hypertension and pre‐eclampsia) in obese women (Song et al., 2023). In their retrospective cohort study, Song et al. (2023). reported that excessive weight gain during the first trimester and rapid weekly weight gain after 20 weeks predicted a high risk of hypertensive disorders in obese pregnant women. They concluded that the first trimester of pregnancy may be critical for gestational weight management (Song et al., 2023).

In the third paper of this section, Yang et al. (2023) describe the relationship between diet quality and excessive GWG from a prospective cohort study (Yang et al., 2023). The study showed that higher diet quality during the second and third trimesters was associated with lower odds of excessive GWG for both normal weight and overweight women. Adequacy of healthy food during the second and third trimesters was protective against excessive GWG (Yang et al., 2023).

In summary, these papers provide evidence that GWG velocity during the first trimester is a key determinant for higher risk of LGA newborns and gestational hypertensive disorders, even though GWG during the second and third trimesters could predict higher risk for maternal outcomes (gestational hypertension and pre‐eclampsia) and birth outcomes (LGA and SGA) as well. The relationship between GWG velocity and other outcomes (e.g., gestational diabetes, post‐partum weight retention and childhood obesity) warrants further exploration. A high‐quality diet during the second and third trimesters might be a protective factor against excessive GWG in the Chinese population. As these studies were mainly conducted in urban China, further studies, especially the association between diet and excessive GWG, and maternal child‐health outcomes, including breastfeeding (Pérez‐Escamilla, 2023), need to be conducted in other parts of China and beyond.

## References

[mcn13597-bib-0001] Goldstein, R. F. , Abell, S. K. , Ranasinha, S. , Misso, M. , Boyle, J. A. , Black, M. H. , Li, N. , Hu, G. , Corrado, F. , Rode, L. , Kim, Y. J. , Haugen, M. , Song, W. O. , Kim, M. H. , Bogaerts, A. , Devlieger, R. , Chung, J. H. , & Teede, H. J. (2017). Association of gestational weight gain with maternal and infant outcomes: A systematic review and meta‐analysis. Journal of the American Medical Association, 317(21), 2207–2225. 28586887 10.1001/jama.2017.3635 PMC5815056

[mcn13597-bib-0002] Goldstein, R. F. , Abell, S. K. , Ranasinha, S. , Misso, M. L. , Boyle, J. A. , Harrison, C. L. , Black, M. H. , Li, N. , Hu, G. , Corrado, F. , Hegaard, H. , Kim, Y. J. , Haugen, M. , Song, W. O. , Kim, M. H. , Bogaerts, A. , Devlieger, R. , Chung, J. H. , & Teede, H. J. (2018). Gestational weight gain across continents and ethnicity: Systematic review and meta‐analysis of maternal and infant outcomes in more than one million women. BMC Medicine, 16(1), 153. 30165842 10.1186/s12916-018-1128-1 PMC6117916

[mcn13597-bib-0003] Harrison, C. L. , Teede, H. , Khan, N. , Lim, S. , Chauhan, A. , Drakeley, S. , Moran, L. , & Boyle, J. (2021). Weight management across preconception, pregnancy, and postpartum: A systematic review and quality appraisal of international clinical practice guidelines. Obesity Reviews, 22(10), e13310. 34312965 10.1111/obr.13310

[mcn13597-bib-0004] IOM (Institute of Medicine) and NRC (National Research Council) . (2009). Weight Gain During Pregnancy: Reexamining the Guidelines. The National Academies Press. 20669500

[mcn13597-bib-0005] Kac, G. , Carilho, T. R. , Rasmussen, K. M. , Reichenheim, M. E. , Farias, D. R. , Hutcheon, J. A. , & Brazilian Maternal and Child Nutrition Consortium . (2021). Gestational weight gain charts: Results from the Brazilian Maternal and Child Nutrition Consortium. The American Journal of Clinical Nutrition, 113(5), 1351–1360. 33740055 10.1093/ajcn/nqaa402 PMC8106749

[mcn13597-bib-0006] Larqué, E. , Labayen, I. , Flodmark, C. E. , Lissau, I. , Czernin, S. , Moreno, L. A. , Pietrobelli, A. , & Widhalm, K. (2019). From conception to infancy—Early risk factors for childhood obesity. Nature Reviews Endocrinology, 15(8), 456–478. 10.1038/s41574-019-0219-1 31270440

[mcn13597-bib-0007] NCD Risk Factor Collaboration . (2016). Trends in adult body‐mass index in 200 countries from 1975 to 2014: A pooled analysis of 1698 population‐based measurement studies with 19.2 million participants. The Lancet, 387(10026), 1377–1396. 10.1016/S0140-6736(16)30054-X PMC7615134 27115820

[mcn13597-bib-0008] Pérez‐Escamilla, R. (2023). Breastfeeding and the obesity pandemic. American Journal of Clinical Nutrition, 118(1), 1–2. 10.1016/j.ajcnut.2023.04.035 37210290

[mcn13597-bib-0009] Santos, S. , Voerman, E. , Amiano, P. , Barros, H. , Beilin, L. , Bergström, A. , Charles, M. A. , Chatzi, L. , Chevrier, C. , Chrousos, G. , Corpeleijn, E. , Costa, O. , Costet, N. , Crozier, S. , Devereux, G. , Doyon, M. , Eggesbø, M. , Fantini, M. , Farchi, S. , … Jaddoe, V. (2019). Impact of maternal body mass index and gestational weight gain on pregnancy complications: An individual participant data meta‐analysis of European, North American and Australian cohorts. BJOG: An International Journal of Obstetrics & Gynaecology, 126(8), 984–995. 30786138 10.1111/1471-0528.15661 PMC6554069

[mcn13597-bib-0010] Song, W. , Zhang, Z. , Zheng, W. , Gao, L. , Liang, S. , Cheng, D. , Wang, X. , Guo, C. , & Li, G. (2023). Patterns of gestational weight gain among women with obesity and its correlation with hypertensive disorders of pregnancy in China. Maternal & Child Nutrition, 19(3), e13416. 36098354 10.1111/mcn.13416 PMC10262902

[mcn13597-bib-0011] Standard of Recommendation for Weight Gain during Pregnancy Period . (2022). *Biomedical and Environmental Sciences*, 35(10), 875. 10.3967/bes2022.114 36443264

[mcn13597-bib-0012] Wang, J. , Li, J. , & Lai, J. (2023). Trajectory of gestational weight gain is related to birthweight: The TAWS cohort study in China. Maternal & Child Nutrition. 10.1111/mcn.13578 PMC11168369 38576191

[mcn13597-bib-0013] Wang, J. , Duan, Y. F. , Pang, X. H. , Jiang, S. , Yin, S. A. , Yang, Z. Y. , & Lai, J. Q. (2018). [Gestational weight gain and optimal ranges in Chinese mothers giving singleton and full‐term births in 2013]. Zhonghua yu fang yi xue za zhi [Chinese Journal of Preventive Medicine], 52(1), 31–37. 29334705 10.3760/cma.j.issn.0253-9624.2018.01.007

[mcn13597-bib-0014] Widen, E. M. , Whyatt, R. M. , Hoepner, L. A. , Ramirez‐Carvey, J. , Oberfield, S. E. , Hassoun, A. , Perera, F. P. , Gallagher, D. , & Rundle, A. G. (2015). Excessive gestational weight gain is associated with long‐term body fat and weight retention at 7 y postpartum in African American and Dominican mothers with underweight, normal, and overweight prepregnancy BMI. The American Journal of Clinical Nutrition, 102(6), 1460–1467. 26490495 10.3945/ajcn.115.116939 PMC4658466

[mcn13597-bib-0015] World Health Organization . (2023). *First global call for data on gestational weight gain*.

[mcn13597-bib-0016] Yang, M. , Feng, Q. , Chen, C. , Chen, S. , Guo, Y. , & Zeng, G. (2023). Healthier Diet associated with reduced risk of Excessive Gestational Weight Gain: A Chinese prospective cohort. Maternal & Child Nutrition. 10.1111/mcn.13397 PMC10262912 35821659

[mcn13597-bib-0017] Zheng, W. , Huang, W. , Zhang, Z. , Zhang, L. , Tian, Z. , Li, G. , & Zhang, W. (2019). Patterns of gestational weight gain in women with overweight or obesity and risk of large for gestational age. Obesity Facts, 12(4), 407–415. 31261149 10.1159/000500748 PMC6758715

